# Successful Management of Recurrent Hemoptysis, Polycythemia and Respiratory Distress in a Dog

**DOI:** 10.3390/ani16091384

**Published:** 2026-04-30

**Authors:** Pin-Yen Chen, Chi-Ru Chen, Po-Yao Huang, Pei-Ying Lo, Wei-Tao Chang, Chung-Hui Lin

**Affiliations:** 1 Lab of Small Animal Respiratory and Cardiovascular Medicine, TACS-Alliance Research Center, Taipei 110, Taiwan; ariel89065@gmail.com (P.-Y.C.); roo201061@gmail.com (C.-R.C.); brandon70534@gmail.com (P.-Y.H.); pylo13@gmail.com (P.-Y.L.); 2 National Taiwan University Veterinary Hospital, National Taiwan University, Taipei 106, Taiwan; 3 Animal Resource Center, National Taiwan University, Taipei 106, Taiwan; 4 Graduate Institute of Veterinary Clinical Sciences, National Taiwan University, Taipei 106, Taiwan

**Keywords:** hemoptysis, erythrocytosis, polycythemia, hemosiderosis, dyspnea, dog

## Abstract

A male castrated Shih Tzu was evaluated for recurrent nocturnal episodes of acute respiratory distress accompanied by hemoptysis and transient erythrocytosis. The dog was clinically normal between episodes, but each nighttime event was severe and prompted repeated emergency visits. During each emergency presentation, thoracic radiographs revealed severe diffuse interstitial-to-alveolar pulmonary infiltrates, and packed cell volume showed marked but reversible increases. A stepwise diagnostic evaluation, including serial indirect blood pressure measurement, coagulation assessment, echocardiography, and bronchoscopy with bronchoalveolar lavage, progressively excluded typical infectious, cardiac, structural, and coagulopathic causes of hemoptysis and acute respiratory distress. Given the stereotyped pattern of near-acute crises with diffuse pulmonary infiltrates and hemoptysis, mechanisms analogous to noncardiogenic pulmonary edema or exercise-induced pulmonary hemorrhage were considered. Therapeutic trials with sildenafil and furosemide failed to prevent further nocturnal recurrences. Considering concurrent transient PCV surges and the proposed role of catecholamine-driven splenic contraction as a rapidly mobilizable erythrocyte reservoir, a sympathetically mediated process was suspected, and α_1_-adrenergic blockade with prazosin was initiated. Following prazosin therapy, sustained clinical remission was achieved, with no further emergency episodes over a 17.5-month follow-up period. The response may have reflected multiple pharmacological effects of prazosin, including attenuation of sympathetically mediated splenic α_1_-adrenergic activity, systemic vasodilation, and reduction in venous return. This unique case suggests that dysregulation of the sympathetic nervous system may have contributed to the recurrent hemoptysis and acute respiratory distress and highlights adrenergic modulation as a potential therapeutic consideration in similar cases.

## Simple Summary

Recurrent hemoptysis with acute respiratory distress is uncommon in dogs and can be challenging to diagnose and manage. This report describes a Shih Tzu dog with repeated midnight emergency visits for severe episodes of respiratory distress with hemoptysis. The dog was clinically normal between episodes, but each nocturnal event was abrupt and severe. During crises, diffuse pulmonary infiltrates on thoracic radiographs, together with transient increases in packed cell volume, were documented. A stepwise diagnostic evaluation excluded typical causes that could lead to hemoptysis and acute respiratory distress. Sequential therapeutic trials failed to prevent further nocturnal recurrences until dysregulated sympathetic activity with splenic contraction was proposed as the underlying mechanism. Long-term clinical stabilization was eventually achieved with α_1_-adrenergic blockade using prazosin. This case highlights an alternative, noninfectious mechanism to consider in dogs with recurrent hemoptysis episodes.

## 1. Introduction

Acute respiratory distress is a common reason for dogs to present to emergency services, accounting for 16% of emergency visits in dogs in a large-scale study [1]. Acute respiratory distress may arise from a broad range of respiratory, cardiovascular, and noncardiopulmonary disorders. However, respiratory distress accompanied by hemoptysis is an uncommon presentation in dogs. Hemoptysis, defined as bleeding originating from the lower respiratory tract, carries a wide differential diagnosis [2,3]. In dogs presenting with hemoptysis, the most commonly reported underlying causes in a retrospective study of 36 cases identified during a 10-year period at a university teaching hospital were bacterial bronchopneumonia (19.4%), respiratory neoplasia (13.9%), and trauma (13.9%), followed by systemic causes such as heartworm disease (11.1%) and immune-mediated thrombocytopenia (11.1%) [2]. Additional reported etiologies associated with hemoptysis or pulmonary hemorrhage include structural or iatrogenic factors (e.g., lung lobe torsion, airway foreign bodies, or bronchoscopic biopsy) [2,4,5], intoxications (most notably anticoagulant rodenticides) [2,6,7], specific infectious agents (e.g., leptospirosis, aspergillosis, or pneumonic plague) [8,9,10,11], and seizure-associated events [3].

Neurogenic mechanisms resulting in acute respiratory distress and, in some cases, hemoptysis have been reported in a limited number of dogs, and the underlying pathophysiology remains incompletely understood [12]. Reported presentations include noncardiogenic pulmonary edema (NCPE) secondary to traumatic brain injury or seizures, as well as hunting-associated acute respiratory distress episodes, for which sympathetic activation has been proposed as a contributing factor [3,13,14]. The present case report describes a *Shih Tzu* with recurrent hemoptysis, episodic erythrocytosis, and acute respiratory distress and discusses inappropriate α-adrenergic stimulation as a possible pathophysiologic mechanism based on the diagnostic evaluation and clinical course.

## 2. Case Description

**Figure 1:**
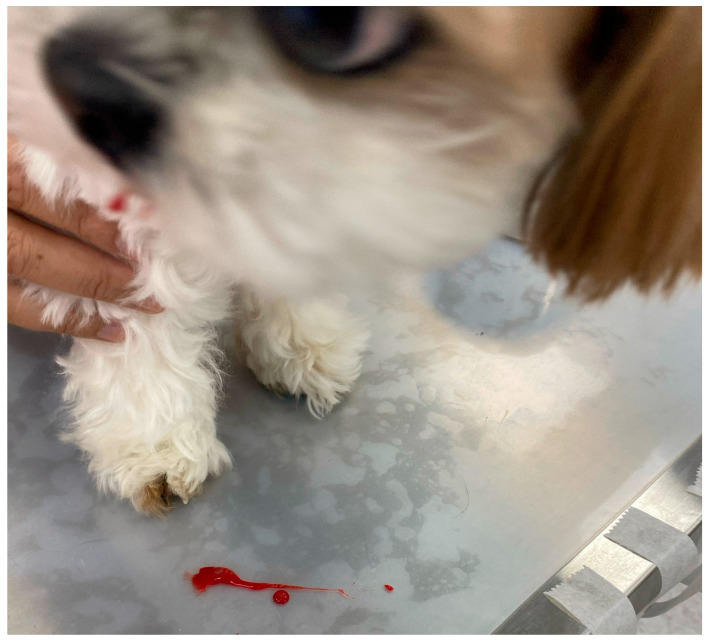
Photograph obtained during the third emergency presentation of this 7-year-old male castrated Shih Tzu for acute respiratory distress. Hemoptysis occurred during the initial physical examination after arrival.

A 7-year-old male castrated *Shih Tzu* was referred for evaluation of recurrent nocturnal emergency visits for acute respiratory distress followed by hemoptysis (Figure 1). A total of five episodes were documented. The first episode occurred when the dog was 6.5 years old. Thereafter, episodes recurred approximately every 4–7 months, with the inter-episode interval gradually shortening to every 2–3 months. All episodes occurred at night after the dog had fallen asleep. Each episode was characterized by sudden awakening from sleep accompanied by marked tachypnea (respiratory rate > 90 breaths per minute). This rapidly progressed to severe orthopnea with loud, harsh, coarse, wet-sounding respiratory noise. Intermittent coughing was also noted. Hemoptysis was observed within 30 min of the onset of each episode. Clinical signs typically resolved within hours following supportive treatment, while hematologic and thoracic radiographic abnormalities normalized within a few days.

**Figure 2:**
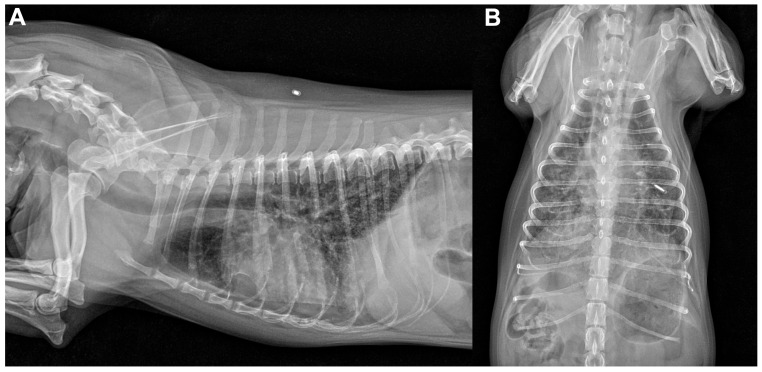
Right lateral (**A**) and ventrodorsal (**B**) thoracic radiographs obtained during one emergency visit show a marked, diffuse interstitial-to-alveolar pattern, most prominent in the caudal lung fields. Radiographic changes during other emergency visits were similar.

During each severe episode requiring emergency admission, marked increases in red blood cell count (RBC), packed cell volume (PCV), and hemoglobin concentration (Hb) were consistently documented at presentation (Table 1), along with a pronounced interstitial-to-alveolar pattern on thoracic radiography (Figure 2). C-reactive protein (CRP) concentrations were measured because of concern for pneumonia/pneumonitis, and elevated CRP concentrations of varying magnitude were documented during some of the emergency visit episodes. The rest of the serum biochemistry (Supplementary File S1) was generally unremarkable, and blood film evaluation did not reveal any abnormalities or evidence of hemoparasites. Testing for heartworm antigen and antibodies to *Ehrlichia* spp., *Anaplasma* spp., and *Borrelia burgdorferi* was negative. At each emergency presentation, pneumonia was tentatively suspected at the initial evaluation, although it did not fully explain all clinical findings, particularly hemoptysis. After the dog received oxygen supplementation via an oxygen cage, fluid therapy with lactated Ringer’s solution, and antimicrobial treatment, including intravenous amoxicillin-clavulanate (20 mg/kg TID) and enrofloxacin (5 mg/kg SID), at the emergency service, PCV and CRP concentrations decreased rapidly over the subsequent 4 days, accompanied by gradual resolution of the radiographic abnormalities.

Between episodes, the dog was clinically normal, with the resting/sleeping respiratory rate consistently <30 breaths per minute and occasional coughing or choking-like clinical signs reported by the owner. The owner also observed intermittent, transient increases in respiratory rate (>50 breaths per minute) during the night upon awakening, although these events were self-limiting and were not clearly confirmed as an overt abnormality. Otherwise, the dog maintained a normal activity level and appetite and did not exhibit noisy breathing or other clinical signs suggestive of upper airway obstruction or other respiratory disease during the inter-episode period. Notably, the dog consistently had a PCV at the high end of the reference interval (>50%), which was present even before the first documented episode of hemoptysis.

As all previous crises occurred in the middle of the night, potential triggers were investigated. A seizure activity or airway obstruction-related event leading to non-cardiogenic pulmonary edema (NCPE) [12] was considered less likely, as the owner did not observe any abnormal movements or an increase in snoring prior to the onset of acute respiratory distress. The owner reported that episodes often seemed to occur the day after a visit to a pet groomer, although this association could not be confirmed as a definitive precipitating event. Differential diagnoses at this stage included recurrent pneumonia secondary to gastroesophageal reflux during sleep, atypical NCPE, and transient pulmonary hypertension of uncertain cause. In addition, systemic hypertension, coagulopathy, and bronchial vascular anomalies predisposing to bleeding could not be ruled out.

Echocardiography indicated a low probability of pulmonary hypertension [15] and stage B1 myxomatous mitral valve disease, findings not supportive of pulmonary hypertension (Supplementary File S1). Electrocardiography was unremarkable.

Serial indirect systolic blood pressure measurements were obtained using Doppler sphygmomanometry (Model 811-B, Parks Medical Electronics, Aloha, OR, USA) in a quiet environment after an acclimation period, with the dog conscious, calm, unsedated, and gently restrained. An appropriately sized cuff, with a width approximately 30–40% of the limb circumference, was placed on the thoracic limb at approximately heart level. For each session, the first measurement was discarded, and the mean of 5–7 consecutive consistent systolic blood pressure measurements was recorded. Across five consecutive visits, the mean systolic blood pressure was approximately 150 mmHg, consistent with a prehypertensive range [16]. Urinalysis revealed a urine protein-to-creatinine ratio of 0.10; therefore, clinically relevant systemic hypertension was considered unlikely as an underlying cause of hemoptysis. Fecal examination was not performed because *Angiostrongylus vasorum* was not endemic in the region. To rule out underlying coagulopathy, prothrombin time and activated partial thromboplastin time were measured prior to referral and were both within the reference range. Therefore, thromboelastography (TEG) was performed to further assess coagulation status. TEG results revealed a mildly reduced maximum amplitude (39.3 mm; reference interval: 42.9–67.9 mm) without evidence of hyperfibrinolysis. However, this clot-strength abnormality was interpreted cautiously because canine viscoelastic coagulation variables, including clot-strength indices, may be influenced by hematocrit/red cell mass and whole-blood viscosity [17,18]. Overall, the abnormality was mild and was considered unlikely to have been a primary contributor to the hemoptysis.

Considering these findings and the dog’s stable clinical condition at presentation, episodic gastroesophageal reflux with secondary pneumonia was prioritized as an initial treatment target. Omeprazole (1 mg/kg, BID), maropitant (0.53 mg/kg, BID), and mosapride (0.35 mg/kg, BID) were administered to reduce suspected gastroesophageal reflux during sleep. However, nocturnal emergency visits for acute respiratory distress followed by hemoptysis continued to recur. At that time, the interval since the previous nocturnal emergency visit was approximately four months.

At this stage, although the clinical presentation did not align with any well-defined etiology in the literature, the recurrent and stereotyped nature of the episodes raised concern for two plausible pathophysiologic mechanisms, given the acute, marked increases in PCV and the consistent presence of hemoptysis during each crisis. The first was α-adrenergic overstimulation, resulting in a transient increase in PCV due to splenic contraction and subsequent pulmonary capillary extravasation. The second was a mechanism loosely analogous to exercise-induced pulmonary hemorrhage (EIPH), a condition that remains incompletely understood and has been reported sporadically in racing horses and greyhounds [19,20]. In contrast to classic EIPH, the present case involved a sleeping dog without intense exertion or large negative intrapleural pressure swings, but the concept that transient hemodynamic loading of the pulmonary microvasculature might predispose to pulmonary capillary stress failure was also considered at this stage. Because several prior episodes appeared to occur on the day following visits to a pet grooming salon, although this association was not confirmed, grooming was tentatively suspected as a potential trigger that might increase pulmonary vascular pressure and was therefore considered within the same broad hemodynamic context. Prior reports have suggested limited efficacy of sildenafil in horses with EIPH and in a dog with hunting-associated respiratory distress [14,21]; therefore, a single dose of sildenafil (2 mg/kg PO) was administered before each grooming session as an attempted preventive trial to mitigate potential increases in pulmonary vascular pressure. Nevertheless, fulminant respiratory distress with hemoptysis still recurred approximately 2 months after the last emergency visit.

**Figure 3:**
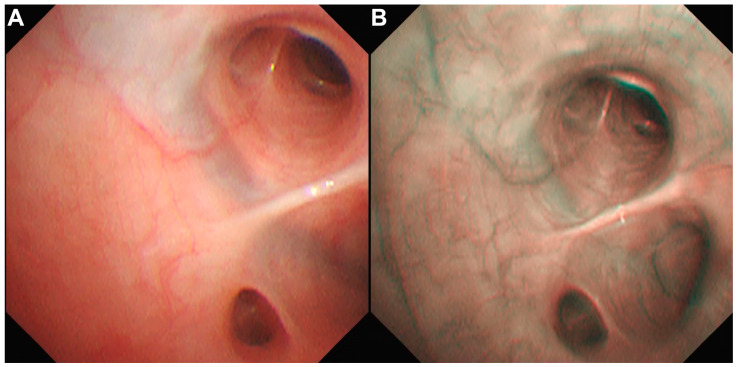
Bronchoscopic examination using white-light imaging (**A**) and narrow-band imaging highlighting superficial blue vessels and submucosal cyan vessels (**B**) revealed no significant vascular anomalies that could explain the recurrent episodes of hemoptysis.

**Figure 4:**
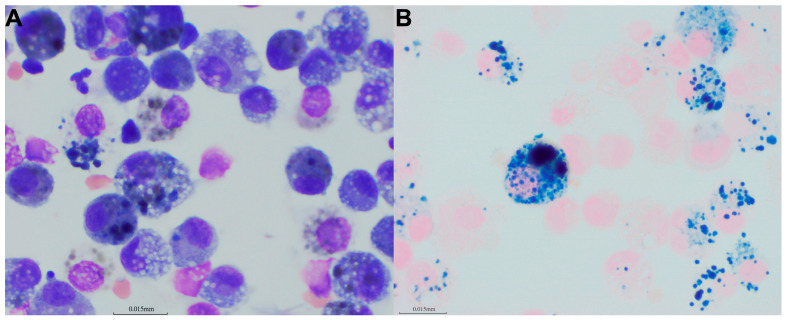
Bronchoalveolar lavage performed 1 week after the fifth emergency visit revealed mild lymphocytosis and abundant, heavily pigmented alveolar macrophages on Giemsa stain (**A**). The pigments were confirmed as hemosiderin with Prussian blue staining (**B**), indicating hemosiderosis, possibly related to the episode of hemoptysis and respiratory distress one week earlier [23]. Images were obtained at 1000× total magnification.

Bronchoscopy with bronchoalveolar lavage (BAL) was pursued as a further diagnostic step, with owner consent, given the progressively shortening intervals between episodes. The procedure was performed one week after the fifth emergency visit to allow for clinical stabilization and to reduce anesthetic risk following recent respiratory distress. Bronchoscopy with BAL was performed to rule out bacterial pneumonia and to assess for bronchial vascular anomalies and residual sanguineous material within the lower respiratory tract. Antimicrobial therapy was discontinued four days prior to the BAL. Bronchoscopic evaluation was performed using a flexible video endoscope (BF-XP290, Olympus; Olympus Co., Tokyo, Japan) under general anesthesia in sternal recumbency, following the canine bronchial map [22]. A recent or recurrent pneumonia was considered unlikely because no mucus accumulation or obvious mucosal irregularities were observed, aside from mild mucosal edema. Cytology, aerobic and anaerobic bacterial culture, fungal culture, and *Mycoplasma* PCR provided no evidence of infection. Common structural abnormalities, including severe bronchomalacia with mild lymphocytic inflammation, grade II tracheal collapse, and mild-to-moderate soft palate elongation, were identified but were not unexpected for this brachycephalic breed. The bronchial vasculature appeared normal on both white-light and narrow-band imaging bronchoscopic examination (Figure 3). BAL fluid was processed using cytospin and stained with Giemsa and Prussian blue. No grossly visible sanguineous material was identified; however, marked hemosiderosis was present (Figure 4), with minimal fresh erythrocytes on BAL fluid analysis.

Based on these findings, recurrent bacterial pneumonia, ulcerative bronchial mucosal lesions, and bronchial vascular anomalies were considered unlikely. An atypical disorder with a mechanism analogous to EIPH or NCPE became the leading suspicion. Because furosemide has been reported as a treatment option for both EIPH and NCPE [12,24], it was prescribed at 1 mg/kg PO q12h, with instructions to administer an additional 4 mg/kg PO on an as-needed basis if nocturnal tachypnea was noted. Although no episodes occurred during the first 2 months after initiating this regimen, acute respiratory distress with hemoptysis recurred and necessitated another emergency visit during the night approximately 4 months after BAL. No antimicrobials were administered during this emergency because the prior BAL had provided no evidence of an infectious etiology; instead, furosemide and judicious intravenous fluid therapy were used as the sole treatments. The dog recovered without antimicrobial therapy, further supporting a noninfectious mechanism. However, although furosemide appeared to improve respiratory distress during acute episodes, it did not prevent recurrence.

With inappropriate splenic contraction secondary to adrenergic stimulation being considered as a potential mechanism underlying the recurrent episodes of hemoptysis, erythrocytosis, and respiratory distress, treatment was directed toward attenuating α-adrenergic overstimulation. Prazosin was initiated in the hospital, and systolic blood pressure was monitored hourly to titrate a dose that would avoid clinically relevant hypotension. Subsequently, prazosin was prescribed at 0.1 mg/kg PO q12h to mitigate the presumed α-adrenergic response, and furosemide was reserved for as-needed administration when tachypnea was suspected. Thereafter, no further episodes requiring emergency intervention have occurred. On follow-up evaluations, systolic blood pressure remained within normal limits, and marked increases in PCV were no longer observed. The dog has remained clinically well, with only one transient nocturnal coughing episode accompanied by tachypnea, which resolved after administration of furosemide (4 mg/kg PO) without the need for an emergency visit. Otherwise, the dog remained bright, active, and alert; the owner also reported occasional, intermittent pink-tinged ocular discharge of unclear clinical significance. Follow-up echocardiography performed 8 months after the last episode demonstrated a low probability of pulmonary hypertension and stage B1 myxomatous mitral valve disease. At the time of writing, 17.5 months have elapsed since the last episode of respiratory distress with hemoptysis, and the dog remains clinically stable.

## 3. Discussion

In the present case, recurrent hemoptysis accompanied by polycythemia was a distinctive feature consistently documented at each nocturnal emergency presentation. Accordingly, a key focus of the diagnostic evaluation was to identify etiologies capable of causing both pulmonary hemorrhage and episodic polycythemia. The initial diagnostic workup, followed by bronchoscopy with BAL, helped exclude an infectious etiology as the primary driver of these events. Although a sympathetically mediated mechanism was considered plausible in this dog, it remained unconfirmed because plasma metanephrine and normetanephrine testing was not available during the clinical course. Ultimately, the stereotyped recurrence pattern, the dog’s responses to sequential therapeutic trials, and insights from the available literature collectively guided the successful clinical management.

Erythrocytosis refers to an increase in circulating red blood cells, typically reflected by increases in PCV and/or hemoglobin concentration. An increased PCV should prompt differentiation between relative and absolute erythrocytosis [25,26,27,28,29]. Absolute erythrocytosis indicates a true expansion of total red blood cell mass and is classified as primary or secondary [26,29]. Primary erythrocytosis is an erythropoietin-independent myeloproliferative disorder, whereas secondary erythrocytosis results from increased erythropoietin production that is either physiologically appropriate (e.g., systemic hypoxemia) or inappropriate, most commonly associated with renal disease or neoplasia [28,30,31]. In contrast, relative erythrocytosis occurs when plasma volume is reduced or when red blood cells are transiently mobilized from storage sites into the circulation [28]. Although respiratory disease may lead to hypoxemia, chronic hypoxic pulmonary disease in dogs rarely induces clinically significant adaptive erythrocytosis, and any increase is typically mild, with PCV values seldom exceeding 65% [32]. In our case, inter-episode PCV values remained within the reference interval but tended to be high-normal, whereas erythrocytosis was observed only during acute events. The most likely explanation for this pattern is transient relative erythrocytosis due to red blood cell redistribution from storage sites, most notably the spleen, rather than a true persistent absolute erythrocytosis.

Despite being atypical, a mechanism analogous to NCPE was considered in this case. NCPE is characterized by accumulation of fluid within the pulmonary interstitium or alveoli in the absence of overhydration or heart failure [12]. Instead, it is associated with increased pulmonary capillary hydrostatic pressure, increased vascular permeability, or a combination of both [12,33,34]. In dogs, documented causes of NCPE include drug-related reactions, airway obstruction, acute respiratory distress syndrome, electrocution, drowning, transfusion-related lung injury, and neurogenic causes [12,34,35,36]. Among these, neurogenic pulmonary edema is regarded as a distinct subtype, and its underlying pathophysiology remains incompletely understood [12,33,34]. One proposed mechanism is that acute central nervous system stimulation may trigger a sudden rise in sympathetic tone with excessive catecholamine release [12,33,37]. This response is thought to induce intense systemic vasoconstriction and redistribution of blood from the high-resistance systemic circulation to the pulmonary vasculature, resulting in an abrupt rise in pulmonary capillary hydrostatic pressure. This hemodynamic shift could impose stress on the alveolar–capillary barrier, allowing protein-rich fluid and even erythrocytes to leak into the alveolar space; clinically, this process has been associated with pink frothy fluid or hemoptysis. This proposed mechanism is commonly referred to as the blast theory, which involves a sudden sympathetic surge and a resultant hemodynamic shift [12,33,36,38]. In the present case, although no overt neurologic insult such as seizures or head trauma was identified, the clinical episodes were repeatedly associated with situations expected to increase sympathetic tone. Therefore, while classic neurogenic pulmonary edema appears unlikely, the underlying mechanism described above may still be relevant in explaining the episodic pulmonary findings observed in this dog.

Although the episodes of recurrent respiratory distress in this dog were exclusively nocturnal, the mechanism of EIPH was taken into consideration in the management of episodic hemoptysis in this case because of the marked hemosiderosis identified in BAL fluid. Typical EIPH is characterized by the presence of blood in the airways following strenuous exertion, a phenomenon extensively documented in Thoroughbred racehorses [20,39,40]. In dogs, evidence of EIPH has also been identified in racing Greyhounds, supported by elevated red blood cell concentrations in BAL fluid following exercise [19]. The fundamental pathogenesis of EIPH is widely accepted to be pulmonary capillary stress failure that occurs when capillary–alveolar transmural pressure exceeds a permeability threshold in the walls of pulmonary capillaries and adjacent alveoli [41]. This transmural pressure is determined by pulmonary capillary pressure and intrapleural pressure and is increased during intense exercise by high pulmonary vascular pressures together with large negative inspiratory intrapleural pressures and exercise-induced hypervolemia driven by sympathetically mediated splenic contraction that releases a massive reserve of erythrocyte-rich blood into the systemic circulation [41]. In contrast to classic EIPH, however, the present case lacked both intense exertion and large negative intrapleural pressure swings. Accordingly, the comparison was intended only to invoke the concept of pulmonary capillary stress failure under transient hemodynamic loading rather than a full mechanistic equivalent of EIPH. Hemodynamic and histological evidence suggests that factors beyond pulmonary arterial hypertension contribute to EIPH, as exercise-induced hypervolemia is strongly associated with hemorrhage severity and small pulmonary vein remodeling with hemosiderin deposition supports a contributory venous component in the development of EIPH lesions [40,41]. The current consensus in horses indicates that only the diuretic furosemide is supported by high-quality evidence for effectively reducing the incidence and severity of EIPH [20]. Furosemide is believed to reduce EIPH in horses by diuresis-related reductions in intravascular volume and capillary stress [24,42], and this rationale led to a trial administration of furosemide in the present case.

Elevated CRP at emergency admission contributed to the clinical suspicion of pneumonia and prompted empirical antimicrobial treatment. However, lower airway sampling and bacterial culture were not performed at the time of emergency respiratory distress because of the dog’s unstable status and the risks associated with general anesthesia and airway manipulation. In dogs, marked CRP elevation has been reported in bacterial and aspiration pneumonia, and values are generally higher than those observed in noninfectious conditions such as cardiogenic pulmonary edema, chronic bronchitis, eosinophilic bronchopneumopathy, and idiopathic pulmonary fibrosis [43,44,45]. Importantly, although CRP can be useful for monitoring treatment response and temporal trends in bacterial or aspiration pneumonia, a high CRP concentration is not synonymous with bacterial infection, and a low or normal concentration does not fully exclude bacterial infection [43,45]. In our case, BAL cytology showed no suppurative inflammation, and culture yielded no bacterial growth, indicating no evidence of bacterial infection or neutrophilic inflammation at the time of sampling. However, bronchoscopy was not performed during the emergency visits, which precluded evaluation of the acute stage and leaves it unclear whether the lungs exhibited a transient inflammatory response during episodes of severe respiratory distress in our dog. Notably, substantial CRP elevations can also occur in noninfectious respiratory diseases, particularly in severe or fatal cases, including canine idiopathic pulmonary fibrosis, acute respiratory distress syndrome, and cardiogenic pulmonary edema [43]. This may help explain the pronounced CRP elevation observed in our case at emergency presentation, given the serious respiratory distress at that time. Although CRP was markedly elevated during several emergency presentations and contributed to the initial clinical suspicion of pneumonia, it is a non-specific marker of inflammation and cannot independently confirm infection. In this case, the absence of cytological or microbiological evidence on subsequent BAL highlights the need for cautious interpretation of CRP values in dogs with acute respiratory distress.

In this case, two pathophysiologic considerations were proposed: α-adrenergic overstimulation and a mechanism analogous to EIPH. Sildenafil was initially administered in an attempt to mitigate pulmonary overperfusion during suspected episodes. Previous work has suggested that sildenafil may reduce pulmonary vascular pressures and potentially attenuate hydrostatic components of edema, and it has been associated with improvement of NCPE in dogs with pulmonary hypertension [46]. Sildenafil has also been proposed as a preventive strategy in humans with swimming-induced pulmonary edema, an episodic condition thought to be driven, at least in part, by elevated pulmonary hydrostatic pressures [47,48]. However, sildenafil appeared to be ineffective in preventing recurrence in the present case. After infectious etiologies and other common causes of episodic pulmonary hemorrhage were considered unlikely, pulmonary capillary stress failure was further suspected. Accordingly, maintenance dosing of furosemide was attempted to reduce the hydrostatic component of edema, given its established use in horses with EIPH [20,24] and reports describing resolution of suspected catecholamine-associated neurogenic pulmonary edema in hunting dogs [13]. While furosemide appeared to facilitate radiographic and clinical improvement during acute events, recurrent episodes of respiratory distress persisted. The canine spleen serves as a sizable, sympathetically controlled erythrocyte reservoir, with up to 30% of the total red cell mass sequestered at rest, which can be rapidly emptied by α-adrenergic stimulation, resulting in a prompt rise in PCV [49,50,51]. Therefore, the marked but transient PCV elevations observed during this dog’s crises are biologically consistent with catecholamine-driven splenic contraction [50,51,52,53]. Comparative physiologic data support the plausibility of the marked transient erythrocytosis observed in this dog. In racing Greyhounds, PCV has been reported to increase from 48% at rest to 62% immediately before racing and to 67% after racing, findings interpreted as being largely consistent with marked splenic contraction, although an additional contribution from plasma volume loss was also considered possible [54]. In this context, the PCV of 71% observed in the present case is of a similar order of magnitude. However, precise estimates were not possible. It remains unclear whether the observed increase in PCV was attributable to splenic contraction alone or whether concurrent hemoconcentration or transient shifts in plasma volume also contributed. Similarly, the degree of pulmonary capillary pressure elevation could not be quantified. Therefore, it could not be determined whether the proposed hemodynamic mechanism was sufficient to cause frank hemorrhage rather than edema alone. Nevertheless, the presence of repeated overt hemoptysis, together with marked hemosiderosis on bronchoalveolar lavage, supports the possibility of hemorrhagic alveolar–capillary leakage rather than edema alone.

The consistently nocturnal and stereotyped timing of the episodes was one of the most distinctive yet incompletely explained features of this case. Although some episodes appeared to occur on the day following visits to a pet grooming salon, this association was not consistently confirmed and does not adequately explain the delayed nighttime onset. Potential but unconfirmed explanations could include sleep-related autonomic fluctuations, positional changes affecting venous return during recumbency, or delayed responses to a preceding daytime trigger such as grooming. For instance, recumbency has been proposed to affect cardiopulmonary dynamics through posture-associated shifts in blood from the lower part of the body or through unfavorable hydrostatic relationships within the pulmonary veins [55]. Furthermore, REM sleep has been associated with a marked increase in sympathetic nerve activity, accompanied by increases in blood pressure and heart rate in human subjects [56]. This REM-related phenomenon may provide a physiologic context for considering sleep-related sympathetic surges as one possible explanation. However, these possibilities remain speculative, and the temporal dissociation between the proposed sympathetic mechanism and the observed nighttime recurrence should be regarded as an unresolved aspect of the present case.

Given the ongoing suspicion of sympathetic overstimulation, the α1-adrenergic antagonist prazosin was considered a reasonable therapeutic option to blunt catecholamine-mediated vasoconstriction and central blood-volume shifts, thereby potentially limiting abrupt increases in pulmonary capillary hydrostatic pressure. A previous report described a hunting dog with exertion-associated respiratory distress and elevated plasma normetanephrine concentrations that was successfully managed with prazosin in combination with atenolol [14]. In human medicine, a comparable process has been described in neurogenic and pheochromocytoma-associated NCPE, in which catecholamine surges and concurrent elevations in PCV have been documented [57]. In the absence of clearly identified triggers for sympathetic overstimulation, the proposed mechanisms remain speculative and should be interpreted with caution. Sympathetic dysregulation could not be biochemically confirmed in this case because metanephrine and normetanephrine concentrations were not measured, as these assays were not available at the emergency services or our institution. This represents one limitation of our proposed interpretation. In addition, prazosin has multiple pharmacological effects beyond splenic α_1_-adrenergic blockade. These include systemic vasodilation and reduced venous return, both of which may decrease preload and lower pulmonary capillary hydrostatic pressure [58]. As such, prazosin may help mitigate abrupt increases in pulmonary capillary pressure and reduce the risk of hydrostatic pulmonary edema, thereby contributing to the observed clinical improvement. Although prazosin therapy was associated with sustained remission, this finding is consistent with, but not confirmatory of, a sympathetically mediated mechanism. If available, measurement of catecholamine metabolites during similar episodes in future cases could be considered as part of a prospective diagnostic approach. This may provide additional objective evidence of sympathoadrenal activation. Further investigation is warranted to clarify the relationship between sympathetic nervous system overstimulation and recurrent pulmonary edema and hemoptysis in dogs.

## 4. Conclusions

This case report describes a dog with a unique presentation of recurrent nocturnal hemoptysis, transient erythrocytosis, and acute respiratory distress that was ultimately managed successfully. The episodic clinical pattern, marked but transient increases in PCV during crises, and sustained long-term control after initiation of α_1_-adrenergic blockade are consistent with a sympathetically mediated pathophysiology as one plausible explanation. Adrenergic modulation may therefore represent a therapeutic consideration in selected dogs with recurrent, unexplained episodes of pulmonary hemorrhage/hemoptysis.

## Figures and Tables

**Table 1:** Blood examination results on day 0 (day of presentation), day 1, and day 5 of the third emergency visit. Marked increases in RBC, PCV, and Hb, along with variably elevated CRP concentrations, were repeatedly observed during emergency episodes, with rapid improvement within days on each occasion.

Variables	Day 0	Day 1	Day 5	Reference Interval
RBC (M/μL)	9.9	7.6	8.4	5.65–8.87
PCV (%)	71.0	51.7	58.0	37.3–61.7
Hb (g/dL)	24.0	18.1	19.9	13.1–20.5
CRP (g/dL)	3.0	82.0	6.0	<10.0

Abbreviations: CRP, C-reactive protein; Hb, hemoglobin; PCV, Packed Cell Volume; RBC, Red blood cell.

## Data Availability

The data presented in this study are available upon request from the corresponding author.

## Supplementary Materials

The following supporting information can be downloaded at: https://www.mdpi.com/article/10.3390/ani16091384/s1. File S1: Hematology and biochemistry results during one of the emergency visits

## Abbreviations

The following abbreviations are used in this manuscript:

BAL	Bronchoalveolar lavage
CRP	C-reactive protein
EIPH	Exercise-induced pulmonary hemorrhage
Hb	Hemoglobin
NCPE	Noncardiogenic pulmonary edema
PCV	Packed cell volume
RBC	Red blood cell
