# Supplementary material for: Successful Management of Recurrent Hemoptysis, Polycythemia and Respiratory Distress in a Dog
Source: Animals (Basel). 2026 Apr 30;16(9):1384. doi: 10.3390/ani16091384 (PMC13162872; doi:10.3390/ani16091384)
Supplement: Supplementary file 1 [file animals-16-01384-s001.zip › animals-4266032-supplementary.pdf]

Supplementary File S1 – Hematology and biochemistry results during one of the emergency visits

Test	Units	Result	Reference interval
Red blood cell	M/uL	**10.54**	5.65–8.87
Hematocrit	%	**72.4**	37.3–61.7
Hemoglobin	g/dL	**24.8**	13.1–20.5
Mean Cell Size	fL	68.7	61.6–73.5
Mean Corpuscular Hemoglobin	pg	23.5	21.2–25.9
Mean Corpuscular Hemoglobin Concentration	g/dL	34.3	32.0–37.9
Reticulocytes	K/µL	75.9	10.0–110.0
White blood cell	K/µL	7.88	5.05–16.76
Neutrophil	K/µL	4.73	2.95–11.64
Lymphocyte	K/µL	2.32	1.05–5.19
Monocyte	K/µL	0.38	0.16–1.12
Eosinophil	K/µL	0.45	0.06–1.23
Basophil	K/µL	0.00	0.00–0.10
Platelet	K/µL	284	148–484
Glucose	mg/dL	99	74–143
Creatinine	mg/dL	1.8	0.5–1.8
Urea	mg/dL	**28**	7–27
Total protein	g/dL	7.7	5.2–8.2
Albumin	g/dL	3.4	2.3–4.0
Globulin	g/dL	5.1	2.8–5.1
Albumin/Globulin ratio		0.79	
Alanine Aminotransferase	U/L	92	10–125
Alkaline Phosphatase	U/L	**10**	23–212
Gamma-glutamyl transferase	U/L	0	0–11
Total bilirubin	mg/dL	0.5	0.0–0.9
Cholesterol	mg/dL	170	110–320
Sodium	mmol/L	151	144-160
Potassium	mmol/L	4.4	3.5–5.8
Chloride	mmol/L	114	109–122
C-reactive protein	mg/dL	**6.5**	0.0–1.0
